# Supplementary material for: Sea lamprey enlightens the origin of the coupling of retinoic acid signaling to vertebrate hindbrain segmentation
Source: bioRxiv. 2023 Jul 7:2023.07.07.548143. Preprint. [Version 1] doi: 10.1101/2023.07.07.548143 (PMC10350081; doi:10.1101/2023.07.07.548143)
Supplement: Supplement 1 [file media-1.pdf]

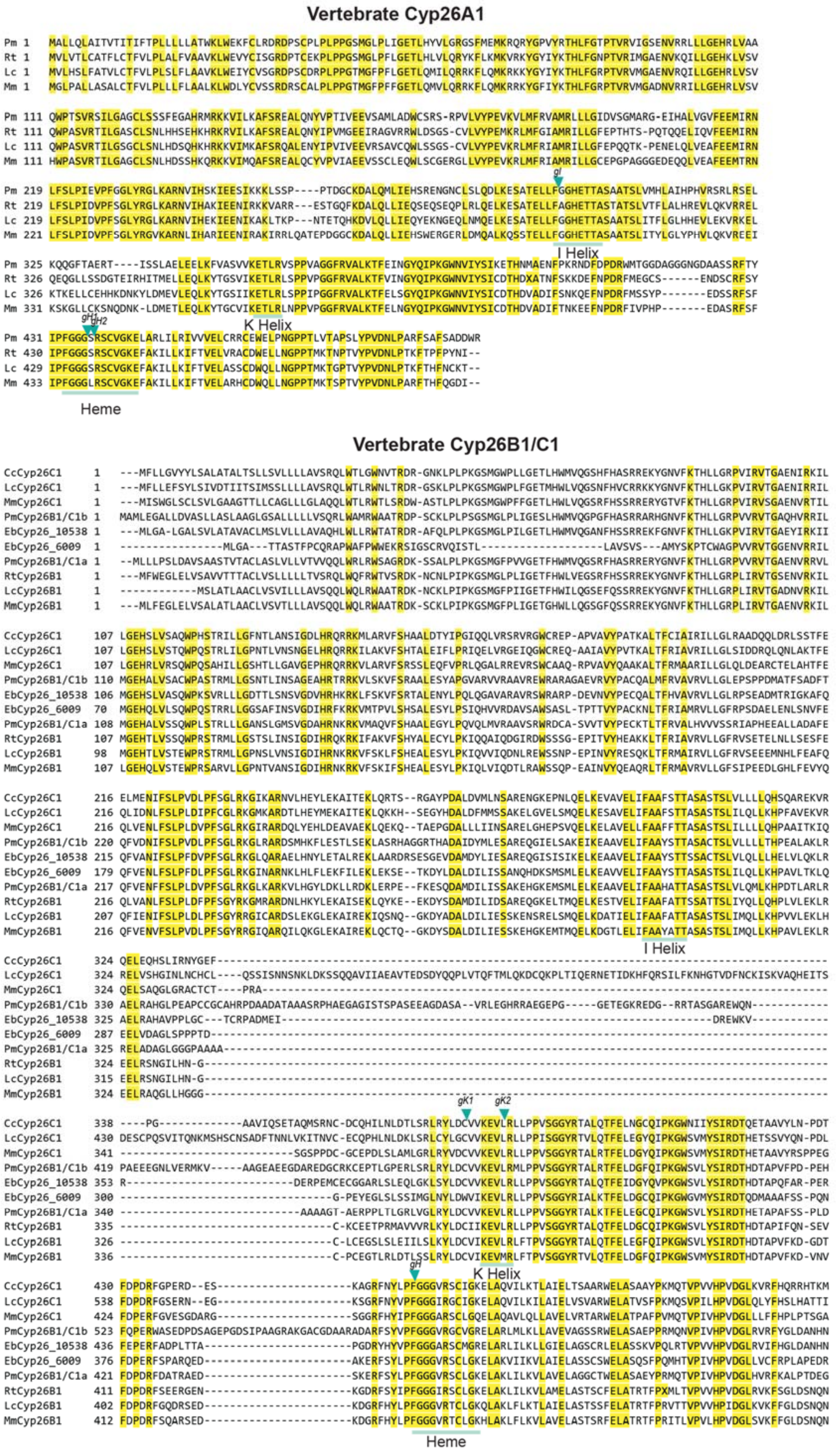
Vertebrate Cyp26A1
I Helix
K Helix
Heme
Vertebrate Cyp26B1/C1
I Helix
K Helix
Heme

Supplementary Figure 1: Vertebrate Cyp26 protein alignments. Protein alignments of vertebrate Cyp26A1 and vertebrate Cyp26B1/C1. The yellow highlighting represents the identity between sequences with a 95% threshold for sequence identity. Conserved K, I helices and Heme domains are indicated. Cutting sites of the gRNAs used for the CRISPR/Cas9 experiments are indicated with a teal arrow. Cc, Carcharodon carcharias; Eb, *Eptatretus burgeri*; Lc, *Latimeria chalumnae*; Mm, *Mus musculus*; Pm, *Petromyzon marinus*; Rt, *Rhincodon typus*.

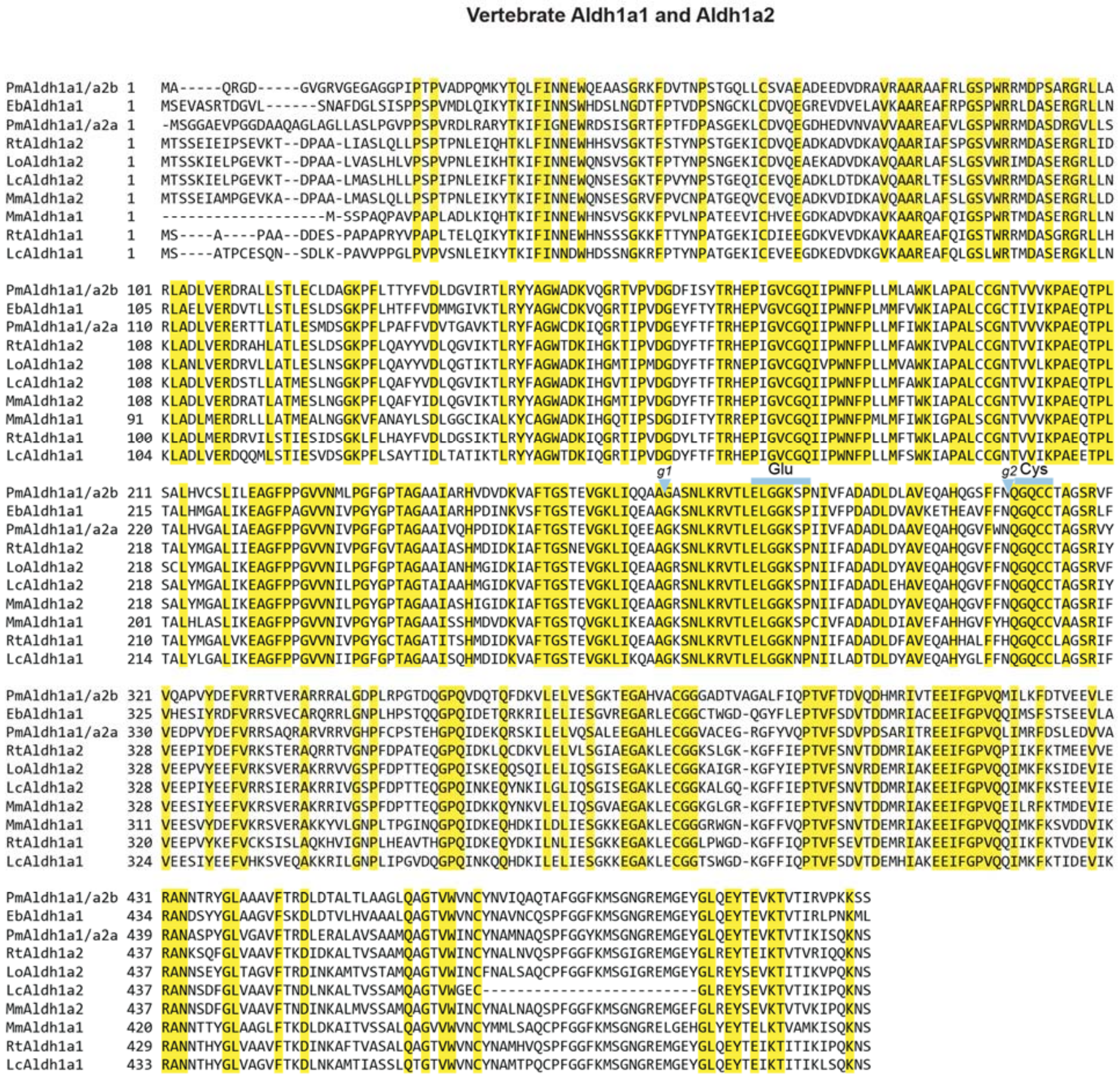

Supplementary Figure 2: Vertebrate Aldh1a protein alignments. Protein alignments of sea lamprey (Pm) Aldh1a1/a2a and Aldh1a1/a2b with Aldh1a1 and Aldh1a2 from various jawed vertebrates. The yellow highlighting represents the identity between sequences with a 95% identity threshold. Conserved Glutamic acid (Glu) and Cysteine (Cys) domains are indicated. Cutting sites of the gRNAs used for the CRISPR/Cas9 experiments are indicated with a blue arrow. Eb, *Eptatretus burgeri;* Lc, *Latimeria chalumnae*; Lo *Lepisosteus oculatus;* Mm, *Mus musculus;* Pm, *Petromyzon marinus*; Rt, *Rhincodon typus.*

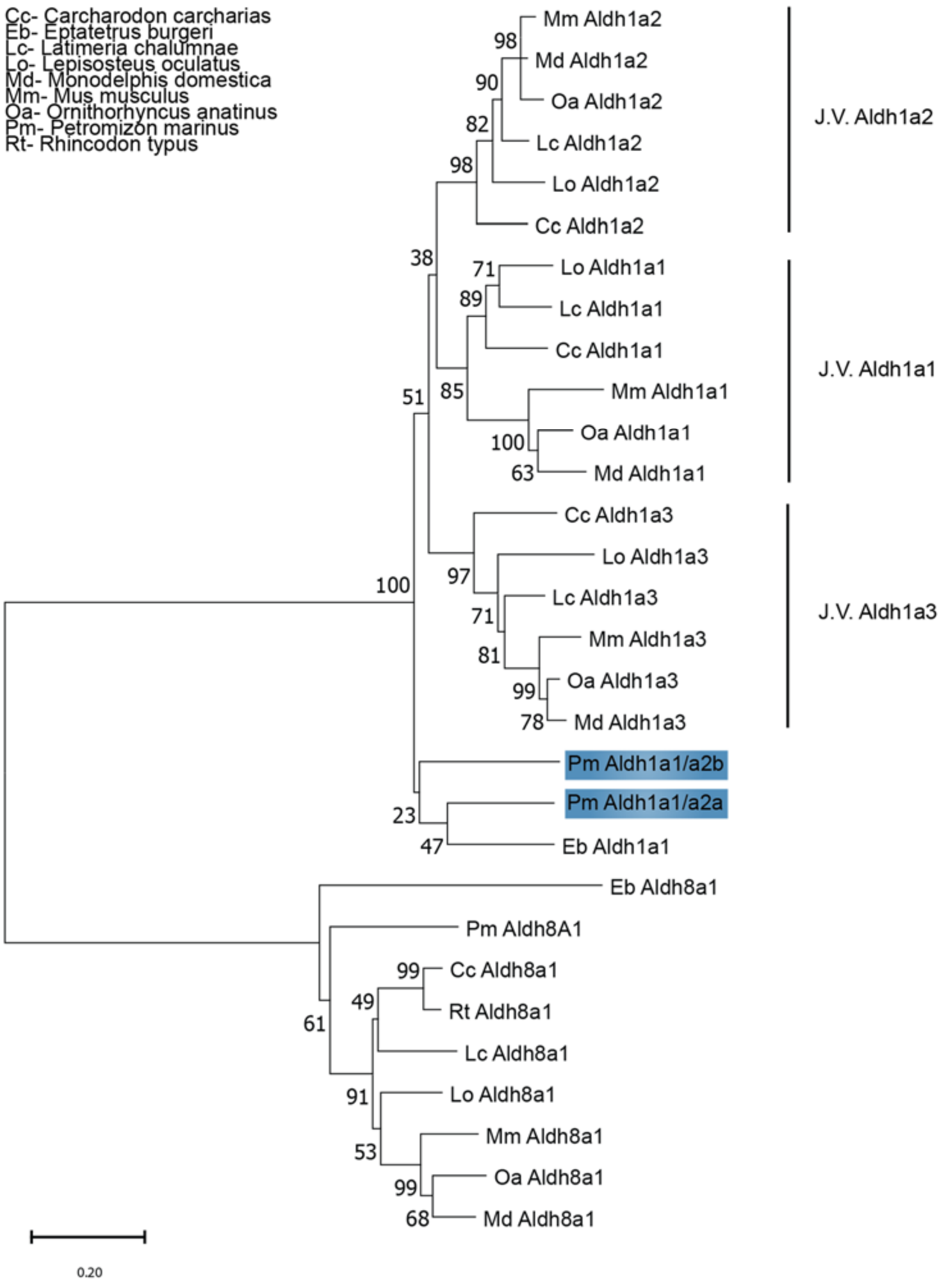

Supplementary Figure 3: Vertebrate *Aldh1a* Phylogeny. Phylogenetic analysis of the vertebrate *Aldh1a* complement with *Aldh1a8* used as an outgroup. Jawed Vertebrate (J.V.) *Aldh1a1/Aldh1a2/Aldh1a3* clades are indicated with black lines. Trees were generated by Maximum Likelihood using the WAG model with 500 iterations for bootstrap testing, and the resulting supporting value for each node is shown as a percentage. A scale bar for the evolutionary distance is indicated below each tree. Species name abbreviations are indicated on the top left of the panel.

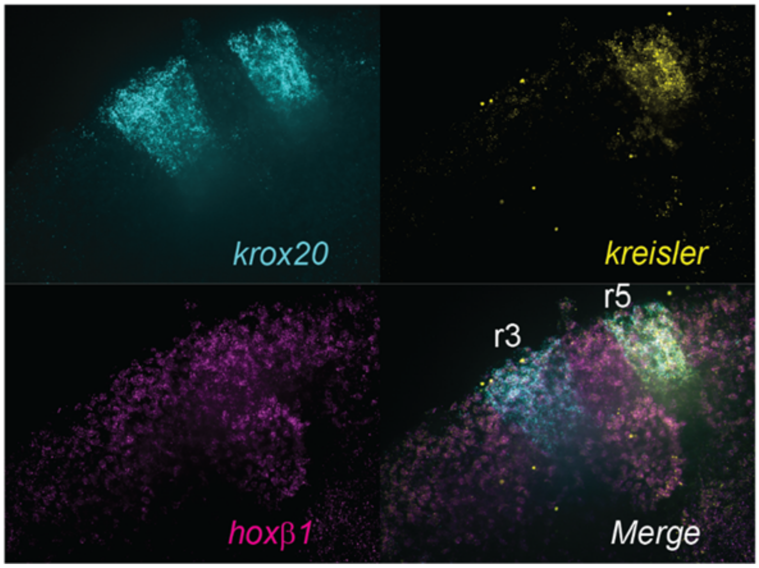

Supplementary Figure 4: HCRv3 development and validation in the sea lamprey. HCR panel representing a high-power (40X) image of *krox20* (cyan), *kreisler* (yellow) and *hoxβ1* (magenta) with their merged expression. This image was obtained after optimizing and adapting our HCRv3 protocol to be able to visualize hindbrain segments. We then used this protocol with this combination of genes to understand the changes in hindbrain segmentation in Talarazole vs. DMSO embryos (**Fig. 6a**).

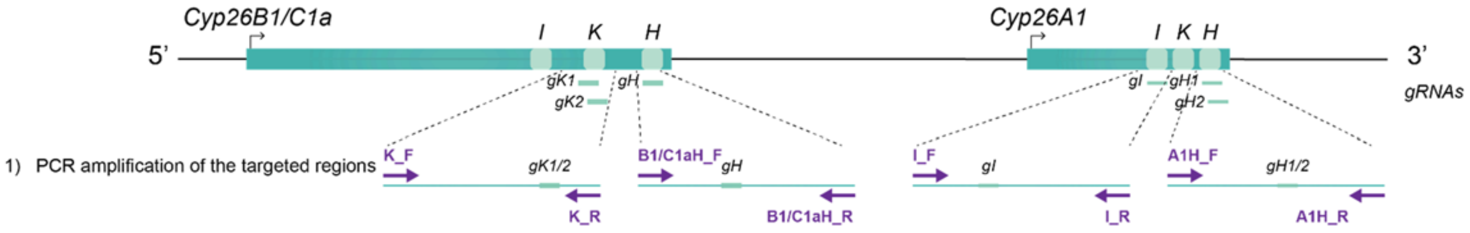

2) Sequencing of the targeted regions

```
Ctrl_C1H     GGAGCGTCCTCTACAGCATCCGCGACACGCACGAGACGGCGCCGGCCTTCAGCTCGCCGC  60
Cr_C1H_1     GGAGCGTCCTCTACAGCATCCGCGACACGCACGAGACGGCGCCGGCCTTCAGCTCGCCGC  60
Cr_C1H_2     GGAGCGTCCTGTACAGCATCCGCGACACGCACGAGACGGCGCCGGCCTTCAGCTCGCCGC  60
Cr_C1H_3     GGAGCGTCCTATACAGCATCCGCGACACGCACGAGACGGCGCCGGCCTTCAGCTCGCCGC  60
Cr_C1H_4     GGAGCGTCCTCTACAGCATCCGCGACACGCACGAGACGGCGCCGGCCTTCAGCTCGCCGC  60
Cr_C1H_5     GGAGCGTCCTCTACAGCATCCGCGACACGCACGAGACGGCGCCGGCCTTCAGCTCGCCGC  60
             ********** *************************************************

Ctrl_C1H     TTGACTTCGACCCCGACCGCTTCGACGCGACGCGCGCCGAGGACTCGAAGGAGCGCTTCA  120
Cr_C1H_1     TTGACTTCGACCCCGACCGCTTAGACGCGACGCGCGCCGAGGACTCGAAGGAGCGCTTCA  120
Cr_C1H_2     TTGACTTCGACCCCGACCGCTTCGACGCGACGCGCGCCGAGGACTCGAAGGAGCGCTTCA  120
Cr_C1H_3     TTGACTTCGACCCCGACCGCTTCGACGCGACGCGCGCCGAGGACTCGAAGGAGCGCTTCA  120
Cr_C1H_4     TTGACTTCGACCCCGACCGCTTCGACGCGACGCGCGCCGAGGACTCGAAGGAGCGCTTCA  120
Cr_C1H_5     TTGACTTCGACCCCGACCGCTTCG------------------------------------  69
             ************************

Ctrl_C1H     GCTACCTGCCGTTCGGCGGCGGCGTGCGCAGCTGCCTGGGCAAGGAGCTCGCCAAGCTCA  180
Cr_C1H_1     GCTACCTGCC----GGCGGCGGCGTGCGCAGCTGCCTGGGCAAGGAGCTCGCCAAGCTCA  176
Cr_C1H_2     GCTACCTGC----CGGCGGCGGCGTGCGCAGCTGCCTGGGCAAGGAGCTCGCCAAGCTCA  176
Cr_C1H_3     GCTACCTGC----CGGCGGCGGCGTGCGCAGCTGCCTGGGCAAGGAGCTCGCCAAGCTCA  176
Cr_C1H_4     GCTACCTGCC----GGCGGCGGCGTGCGCAGCTGCCTGGGCAAGGAGCTCGCCAAGCTCA  176
Cr_C1H_5     ---------------GCGGCGGCGTGCGCAGCTGCCTGGGCAAGGAGCTCGCCAAGCTCA  129
                            *********************************************


Ctrl_A1H     CCCGACCGCTGGATGACCGGCGGCGACGCCGGCGGCGGCAACGGAGACGCGGCATCGTCG  120
Cr_A1H_1     CCCGACCGCTGGATGACCGGCGGCGACGCCGGCGGCGGCAACGGAGACGCGGCATCGTC-  119
Cr_A1H_2     CCCGACCGCTGGATGACCGGCGGCGACGCCGGCGGCGGCAACGGAGACGCGGCATCGTCG  120
Cr_A1H_3     CCCGCCCGCGGGATGACCGGCGGCGCGGCCTGCGGCGGCATCGGAGACGTGGCATTGTCG  120
Cr_A1H_4     CCCGACCGCTGGATGACCGGCGGCGACGCCGGCGGCGGCAACGGAGACGCGGCATCGTC-  119
             **** **** ***************  *** ********* ******** ***** ***

Ctrl_A1H     CGCTTCACCTACATCCCGGGGGCGGCTCGCGCAGCTGCGTGGGCAAGGAGCTGGCGCGAC  180
Cr_A1H_1     ----------------------------GCGCAGCTGCGTGGGCAAGGAGCTGGCGCGAC  151
Cr_A1H_2     CGCTTCACCTCG--------------------CGCTGCGTGGGCAAGGAGCTGGCGCGAC  160
Cr_A1H_3     CGCGTCTC--------------------CTCCCGCTGCGTGGGCAAGGAGCTGGCGCGAC  160
Cr_A1H_4     ----------------------------GCGCAGCTGCGTGGGCAAGGAGCTGGCGCGAC  151
                                               **************************

Ctrl_A1H     TCATCCTGAGGATCGTGGTGGTGGAGCTGTGCCGGCGCTGCGAGTGGGAGCTGCCCAACG  240
Cr_A1H_1     TCATCCTGAGGATCGTGGTGGTGGAGCTGTGCCGGCGCTGCGAGTGGGAGCTGCCCAACG  211
Cr_A1H_2     TCATCCTGAGGATCGTGGTGGTGGAGCTGTGCCGGCGCTGCGAGTGGGAGCTGCCCAACG  220
Cr_A1H_3     TCATCCTGAGGATCGTGGTGGTGGAGCTGTGCCGGCGCTGCGAGTGGGAGCTGCCCAACG  220
Cr_A1H_4     TCATCCTGAGGATCGTGGTGGTGGAGCTGTGCCGGCGCTGCGAGTGGGAGCTGCCCAACG  211
             ************************************************************

Ctrl_A1I     CCGCTCACCACTCCCGCCCCCTCCAGGACCTGAAGGAGTCGGCCACGGAGCTCCTGTTCG  420
Cr_A1I       CCGCTCACCACTCCCGCCCCCTCCAGGACCTGAAGGAGTCGGCCACGGAGCTCCTGT--G  419
             *********************************************************  *

Ctrl_A1I     GGGGACACGAGACGACGGCCAGCGCCGCCACGTCACTCGTCATGCACCTGGCAATCCACC  480
Cr_A1I       GGGGACACGAGACGACGGCCAGCGCCGCCACGTCGCTCGTCATGCACCTGGCAATCCACC  478
             ********************************** *************************
```

	Cyp26A1_H	Cyp26A1_I	Cyp26B1/C1a_H
Mutant CRISPR loci	4/7	1/12	5/6
Control CRISPR loci	5/5	6/6	3/3

Supplementary Figure 5: **Genotyping strategy and sequencing results for *Cyp26* CRISPR embryos**
Cartoon illustrating the design of primers for amplifying each site targeted by the *gRNAs* in the *Cyp26* loci. The primer binding sites are indicated in bold purple. For each site, we show an alignment of mutant loci (Cr_) sequences with the sequence of a control locus (Ctrl), the site targeted by the gRNA is indicated in bold and the PAM in red. A table indicating the proportion of mutant CRISPR or control CRISPR loci for each site is shown.

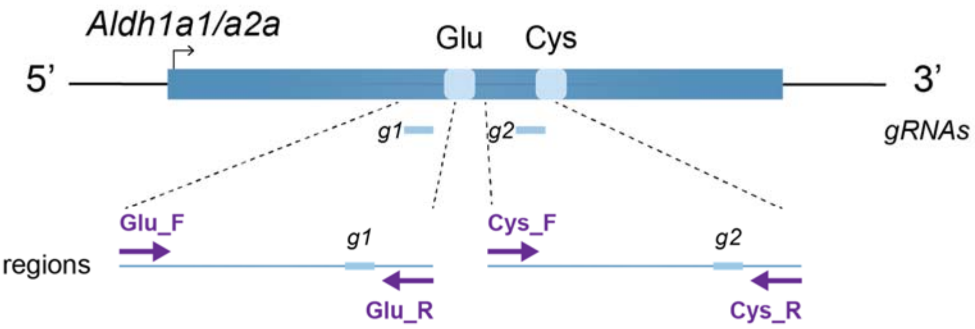

1) PCR amplification of the targeted regions

2) Sequencing of the targeted regions

```
Ctrl_Glu    CCCTTACGTAACGCAGGTGGGGAAGTTGATCCAGGAGGAGGCCGGCAAGAGTAACCTGAA   179
Cr_Glu_1    CCTTTACGTAACGCAGGTGGGGAAGTTGATCCAGGA--------------GTAACGTGAA   73
Cr_Glu_2    CCCTTACGTAACGCAGGTGGGGAAGTTGATCCAGGA--------------GTAACCTGAA   165
Cr_Glu_3    CCCTTACGTAACGCAGGTGGGGAAGTTGATCCAGGA--------------GTAACCTGAA   166
Cr_Glu_4    CCCTTACGTAACGCAGGTGGGGAAGTTGATCCAGGAGTAACCTGAAGC------------   165
Cr_Glu_5    CCCTTACGTAACGCAGGTGGGGAAGTTGATCCAGGA--------------GTAACCTGAA   165
            ** *********************************

Ctrl_Glu    GCGCGTGACGCTGGAGCTTGGCGGGAAGAGCCCCATCATCGTCTTCGCCGACGCCGACCG   239
Cr_Glu_1    GCGAGTGACGCTGAAGCTTGGCGGGAAGAGCCCCATCATCGTATTCGCCGAA-CCGACCG   132
Cr_Glu_2    GCGCGTGACGCTGGAGCTTGGCGGGAAGAGCCCCATCATCGTCTTCGCCGACGCCGACCG   225
Cr_Glu_3    GCGCGTGACGCTGGAGCTTGGCGGGAAGAGCCCCATCATCGTCTTCGCCGACGCCGACCG   226
Cr_Glu_4    ---GCGGACGCTGGAGCGTGGCGGGAAGAGCCCCATCATCGTTTTGACCGACGCCGACCG   222
Cr_Glu_5    GCGCGTGACGCTGGAGCTTGGCGGGAAGAGCCCCATCATCGTCTTCGCCGACGCCGACCG   225
                  ******* *** ************************ **  ****  *******


Ctrl_Cys    CCCCTGCTGTGACACTGCTGACTCGGCGTGGTTCCCACCCCTCCACCCCCCCCCTCCCAC   240
Cr_Cys_1    CCCCTGCTGTGACACTGCTGACTCGGCGTGGTTCCCACCCCTCCACCCCCCCCCTCCCAC   240
Cr_Cys_2    CCCCTGCTGTGACACTGCTGACTCGGCGTGGTTCCCACCCCTCCACCCCCCCCCTCCCAC   240
Cr_Cys_3    CCCCTGCTGTGACACTGCTGACTCGGCGTGGTTCCCACCCCTCCACCCCCCCC-TCCCAC   240
Cr_Cys_4    CCCCTGCTGTGACACTGCTGACTCGGCGTGGTTCCCACCCCTCCACCCCCCCCCTCCCAC   238
Cr_Cys_5    CCCCTGCTGTGACACTGCTGACTCGGCGTGGTTCCCACCCCTCCACCC------------   238
Cr_Cys_6    CCCCTGCTGTGACACTGCTGACTCGGCGTGGTTCCCACCCCTCCACCC------------   238
Cr_Cys_7    CCCCTGCTGTGACACTGCTGACTCGGCGTGGTTCCCACCCCTCCACCC------------   240
            ************************************************

Ctrl_Cys    TACGAGCAGTGGACGCGGCGGTGGAGCAGGCGCACCAGGGGGTGTTCTGGAACCAGGGCC   300
Cr_Cys_1    -------------------------------------------------------GGGCC   258
Cr_Cys_2    -------------------------------------------------------GGGCC   258
Cr_Cys_3    -------------------------------------------------------GGGCC   249
Cr_Cys_4    -------------------------------------------------------GGGCC   260
Cr_Cys_5    ------------------------------------------------------AGGGCC   250
Cr_Cys_6    ------------------------------------------------------AGGGCC   250
Cr_Cys_7    ------------------------------------------------------AGGGCC   244
                                                                   *****
```

	Aldh1a1/a2a_Glu	Aldh1a1/a2a_Cys
Mutant CRISPR loci	5/5	7/11
Control CRISPR loci	5/5	3/3

Supplementary Figure 6: **Genotyping strategy and sequencing results for *Aldh1a1/a2a* CRISPR embryos.** Cartoon illustrating the design of primers for amplifying each site targeted by the *gRNAs* in the *Aldh1a1/a2a* gene loci. Primer binding sites are indicated in bold purple. For each site, we show an alignment of mutant loci (Cr_) sequences with the sequence of a control locus (Ctrl), the site targeted by the gRNA is indicated in bold and the PAM in red. A table indicating the proportion of mutant CRISPR or control CRISPR loci for each site is shown.

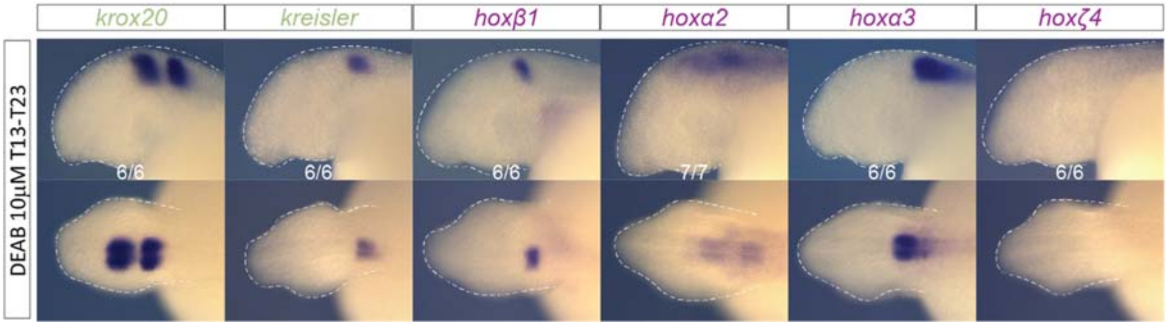

Supplementary Figure 7: **Effect of lower concentration of DEAB on hindbrain patterning.** cISH of key patterning hindbrain markers in embryos treated with 10 µM of DEAB at gastrulation (st13). For each gene, the most representative phenotype is shown, and numbers of experimental replicate are indicated. These molecular phenotypes are less severe than the ones obtained when treating the embryos with 50 µM DEAB (**Fig. 5d**) and are reminiscent of the phenotypes obtained in the Cr *Aldh1a1/a2a* mutants (**Fig. 8c**).

Mouse (*Mus musculus*): Aldh1a1: NP_001348432.1; Aldh1a2: NP_033048.2; Aldh8a1: NP_848828.1; Cyp26A1: XP_017173542.1; Cyp26B1: NP_001171184.1; Cyp26C1: XP_017173748.1; Cyp51: NP_064394.2

Grey short-tailed opossum (*Monodelphis domestica*): Aldh1a1: XP_001373128.2; Aldh1a2: XP_007479744.1; Aldh8a1: NP_848828.1; Cyp26A1: XP_001375292.2; Cyp26C1: XP_007478860.1; Cyp51-like: XP_001378190.2

Platypus (*Ornithorhynchus anatinus*): Aldh1a1: XP_007665209.1; Aldh1a2: XP_028925883.1; Aldh8a1: XP_001509667.1; Cyp26B1: XP_028902395.1; Cyp26C1: XP_028916075.1; Cyp51: XP_028926815.1

Spotted gar (*Lepisosteus oculatus*): Aldh1a1: XP_015222024.1; Aldh1a2: XP_006628784.1; Aldh8a1: XP_006625886.1; Cyp26A1-LG5: XP_015202021.1; Cyp26B1-LG5: XP_015202022.1; Cyp26B1-LG4: XP_006629157.1; Cyp51: XP_006636075.1

West indian coelacanth (*Latimeria chalumnae*): Aldh1a1: XP_006001725.2; Aldh1a2: XP_005998797.2; Aldh8a1: XP_014352052.1; Cyp26A1: XP_005991616.1; Cyp26B1: XP_005997719.1; Cyp26C1: XP_014341348.1; Cyp51: XP_014350652.1

Whale shark (*Rhincodon typus*): Aldh1a1-like: XP_015222024.1; Aldh1a2: XP_020369553.1; Aldh8a1: XP_048452951.1; Cyp26A1: XP_020382659.1; Cyp26B1: XP_020378695.1; Cyp26C1: XP_020367038.1; Cyp51: XP_020371022.1

Inshore hagfish (*Eptatretus burgeri*): Aldh1a1: ENSEBUG00000010361; Aldh8a1: ENSEBUG00000011145; Cyp26C1 (10538): ENSEBUG00000010538; Cyp26C1 (06909): ENSEBUG00000006909; Cyp51: ENSEBUG00000006843

Sea lamprey (*Petromyzon marinus*): Aldh1a1/a2a (Chr1): XP_032805864.1; Aldh1a1/a2b (Ch40): XP_032824682.1; Aldh8a1: XP_032810842.1; Cyp26A1: XP_032808019.1-2; Cyp26B1/C1a (Chr11): XP_032808017.1; Cyp26B1/C1b (Chr9): XP_032806163.1; Cyp51: XP_032816590.1

Supplementary Figure 8: Protein accession numbers used for phylogeny analysis. Protein sequences corresponding to each gene were retrieved using both ENSEMBL and NCBI databases, focusing on the latest available version of the genome and/or the most complete available genome annotation. Cyp51 and Aldh8a1 gene families were used as outgroups for the analyses of Cyp26 and Aldh1a complements, respectively.

Mouse (*Mus musculus*): *Aldh1a1*: NM_001361503.1; *Aldh1a2*: NM_009022.4; *Cyp26A1*: XM_017318053.2; *Cyp26B1*: NM_001177713.1; *Cyp26C1*: XM_017318259.3

Grey short-tailed opossum (*Monodelphis domestica*): *Aldh1a1*: XM_001373091.4; *Aldh1a2*: XM_007479682.2; *Cyp26A1*: XM_001375255.4; *Cyp26C1*: XM_007478798.1

Platypus (*Ornithorhynchus anatinus*): *Aldh1a1*: XM_007667019.3; *Aldh1a2*: XM_029070050.1; *Cyp26B1*: XM_029046562.2; *Cyp26C1*: XM_029060242.1

Chicken (*Gallus gallus*): *Aldh1a1*: NM_204577.5; *Aldh1a2*: NM_001397808.1; *Cyp26A1*: XM_015288598.4; *Cyp26B1*: XM_015286068.4; *Cyp26C1*: XM_421678.8

Spotted gar (*Lepisosteus oculatus*): *Aldh1a1*: XM_015366538.1; *Aldh1a2*: XM_006628721.2; *Cyp26A1LG5*: XM_015346535.1; *Cyp26B1-LG5*: XM_015346536.1; *Cyp26B1-LG4*: XM_006629094.2

West indian coelacanth (*Latimeria chalumnae*): *Aldh1a1*: XM_006001663.2; *Aldh1a2*: XM_005998735.2; *Cyp26A1*: XM_005991554.1; *Cyp26B1*: XM_005997657.2; *Cyp26C1*: XM_014485862.1

Whale shark (*Rhincodon typus*): *Aldh1a1-like*: XM_020527364.2; *Aldh1a2*: XM_020513964.2; *Cyp26A1*: XM_048608684.1; *Cyp26B1*: XM_020523106.1; *Cyp26C1*: XM_020511449.1

Sea lamprey (*Petromyzon marinus*): *Aldh1a1/a2a* (Chr1): XM_032949973.1; *Aldh1a1/a2b* (Chr40): XM_032968791.1; *Cyp26A1*: XM_032952128.1; *Cyp26B1/C1a* (Chr11): XM_032952126.1; *Cyp26B1/C1b* (Chr9): XM_032950272.1

Supplementary Figure 9: mRNA accession numbers used for synteny analysis. mRNA accession numbers of *Cyp26* and *Aldh1a* used to conduct the synteny analyses in different vertebrate models.

Species	Exons	AA
Pm Cyp26A1	7 exons	497 AA
Pm Cyp26B1/C1a	6 exons	529 AA
Pm Cyp26B1/C1b	6 exons	634 AA
Eb Cyp26 06909	6 exons	468 AA
Eb Cyp26 10538	6 exons	528 AA

Supplementary Table 1: Cyp26 gene structure and protein length in cyclostomes. Pm, *Petromyzon marinus* (sea lamprey); Eb, *Eptatretus burgeri* (inshore hagfish); AA. Amino-acid.

Species	***Cyp26A1***	**Cyp26A1**	***Cyp26B1***	**Cyp26B1**	***Cyp26C1***	**Cyp26C1**
Mm	7 exons	497 AA	6 exons	512 AA	6 exons	518 AA
Oa	NF		6 exons	511 AA	6 exons	571 AA
Lo	7 exons	492 AA	6 exons	511 AA	6 exons	554 AA
Lc	7 exons	493 AA	6 exons	511 AA	6 exons	634 AA
Rt	7 exons	494 AA	6 exons	511 AA	NF	
Cc					6exons	560 AA

Supplementary Table 2: Cyp26 gene structure and protein length in jawed vertebrates. Mm, *Mus musculus* (mouse); *Oa, Ornithorhynchus anatinus* (platypus)*; Lo, Lepisosteus oculatus* (spotted gar)*;* Lc, *Latimeria chalumnae* (coelacanth); Rt, *Rhincodon typus* (whale shark); *Cc, Carcharodon carcharias* (great white shark); NF: not found; AA. Amino-acid.

Species	Exons	AA
Pm Aldh1a1/a2a	13	520 AA
Pm Aldh1a1/a2b	13	512AA or 459 AA
Eb Aldh1a1	13	515 AA

Supplementary Table 3: Aldh1a gene structure and protein length in cyclostomes. Pm, *Petromyzon marinus* (sea lamprey); Eb, *Eptatretus burgeri* (inshore hagfish); AA. Amino-acid.

Species	***Aldh1a1***	**Aldh1a1**	***Aldh1a2***	**Aldh1a2**
Mm	13 exons	501 AA	13 exons	518 AA
Oa	13 exons	511 AA	13 exons	518 AA or 422 AA
Lo	13 exons	519 AA or 527 AA	13 exons	518 AA
Lc	13 exons	514	13 exons	518 AA or 422 AA
Rt	13 exons	510 or 537 AA	13 exons	518 AA

Supplementary Table 4: Aldh1a gene structure and protein length in jawed vertebrates. Mm, *Mus musculus* (mouse); *Oa, Ornithorhynchus anatinus* (platypus); *Lo, Lepisosteus oculatus* (spotted gar); Lc, *Latimeria chalumnae* (coelacanth); Rt, *Rhincodon typus* (whale shark); AA. Amino-acid.

Gene	Accession Number	Probe set size	Amplifier/AlexaFluor	Average Probe Concentration
Aldh1a1/a2a	LOC116940317	19	B3-488	6 nM
Cyp26A1	LOC116941264	23	B4-546	5 nM
Cyp26B1/C1a	LOC116941263	22	B1-647	5 nM
krox20 (egr1)	LOC116940673	18	B3-488	6 nM
kreisler (mafB-like)	LOC116954108	12	B4-546	11 nM
hoxβ1	LOC103091820	13	B1-647	8 nM

Supplementary Table 5: HCR FISH probe set design. Summary of the probe set design obtained for each gene targeted by FISH HCR; nM: nanomolar.

name	5'--> 3' sequence	description	product size
A1_H_F	TACAGCATCAAGGAGACGCA	Cyp26A1_H helix	405 bp
A1_H_R	CGTCTTGTGCTGGCATAAATG		
A1_I_F	GCTGAGACAAAGCGGGAAAG	Cyp26A1_I helix	568 bp
A1_I_R	CCAGGCGAGGGAGATAACAT		
**name**	**5'--> 3' sequence**	**description**	**product size**
C1_H_F	GGAGCGTCCTCTACAGCATC	Cyp26C1_H helix	480 bp
C1_H_R	CTCCTCCGTCTCTCTGTCGT		
**name**	**5'--> 3' sequence**	**description**	**product size**
Glu_F	TGGCGGTCGATTCTTCTCTT	Glutamate site genotyping	360 bp
Glu_R	TATTGGCTGCGCAATCTCAC		
Cys_F	ATATACAGCAGGCGTCAGGG	Cysteine site genotyping	355 bp
Cys_R	CGCACGAACTCGTCGTAGA		

Supplementary Table 6: Primers for CRISPR genotyping. Primers used for genotyping targeted regions in CRISPR embryo; F: Forward, R: Reverse.
